# Diethyl *N*,*N*′-(*p*-phenylene)dioxamate

**DOI:** 10.1107/S1600536808027190

**Published:** 2008-08-30

**Authors:** Wei Yang, Xiaoyu Liu

**Affiliations:** a School of Life Sciences, Northeast Normal University, Changchun 130024, People’s Republic of China; b College of Sciences, Shenyang Agricultural University, Shenyang 110161, People’s Republic of China

## Abstract

In the crystal structure, the mol­ecule of the title compound, C_14_H_16_N_2_O_6_, is located on an inversion centre. The amide –NHCO– plane makes a dihedral angle of 34.08 (9)° with the benzene ring. The mol­ecules are connected *via* inter­molecular O—H⋯N hydrogen bonds into a two-dimensional network parallel to the *bc* plane. An intramolecular N—H⋯O hydrogen bond is also observed.

## Related literature

For related literature, see: Hashmi *et al.* (2004 ▶); Navarro *et al.* (1998 ▶); Nonoyama *et al.* (1982 ▶); Pardo *et al.* (2003 ▶); Rios-Moreno *et al.* (2003 ▶).
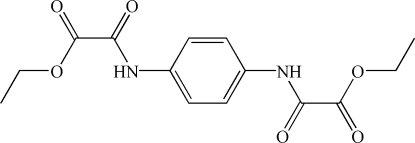


## Experimental

### 

#### Crystal data

C_14_H_16_N_2_O_6_
*M* *_r_* = 308.29
Monoclinic, 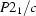

*a* = 11.328 (5) Å
*b* = 7.769 (5) Å
*c* = 8.372 (5) Å
β = 95.566 (5)°
*V* = 733.3 (7) Å^3^
*Z* = 2
Mo *K*α radiation
μ = 0.11 mm^−1^
*T* = 295 (2) K
0.3 × 0.2 × 0.1 mm

#### Data collection

Bruker APEXII CCD area-detector diffractometer
Absorption correction: multi-scan (**SADABS**; Sheldrick, 2003 ▶) *T*_min_ = 0.975, *T*_max_ = 0.989
5037 measured reflections
1285 independent reflections
1075 reflections with *I* > 2σ(*I*)
*R*_int_ = 0.017

#### Refinement

*R*[*F* ^2^ > 2σ(*F* ^2^)] = 0.048
*wR*(*F* ^2^) = 0.136
*S* = 1.00
1285 reflections
104 parameters
H atoms treated by a mixture of independent and constrained refinement
Δρ_max_ = 0.33 e Å^−3^
Δρ_min_ = −0.19 e Å^−3^

### 

Data collection: *APEX2* (Bruker, 2004 ▶); cell refinement: *APEX2*; data reduction: *APEX2*; program(s) used to solve structure: *SHELXS97* (Sheldrick, 2008 ▶); program(s) used to refine structure: *SHELXL97* (Sheldrick, 2008 ▶); molecular graphics: *SHELXTL-Plus* (Sheldrick, 2008 ▶); software used to prepare material for publication: *SHELXL97*.

## Supplementary Material

Crystal structure: contains datablocks global, I. DOI: 10.1107/S1600536808027190/is2327sup1.cif

Structure factors: contains datablocks I. DOI: 10.1107/S1600536808027190/is2327Isup2.hkl

Additional supplementary materials:  crystallographic information; 3D view; checkCIF report

## Figures and Tables

**Table 1:** Hydrogen-bond geometry (Å, °)

*D*—H⋯*A*	*D*—H	H⋯*A*	*D*⋯*A*	*D*—H⋯*A*
N1—H1*N*⋯O2	0.85 (2)	2.30 (2)	2.701 (3)	109.0 (19)
N1—H1*N*⋯O3^i^	0.85 (2)	2.21 (3)	3.030 (3)	161 (2)

Symmetry code: (i) 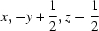.

**Fig. 1.**
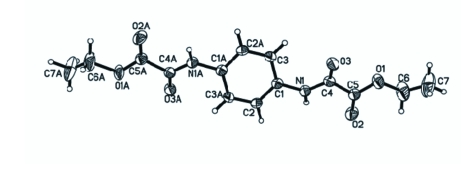
The molecular structure of the title compound, with the unique atom-labelling scheme. The suffix A corresponds to symmetry code (-x + 2, -y, -z + 2). Displacement ellipsoids are drawn at the 30% probability level.

**Fig. 2.**
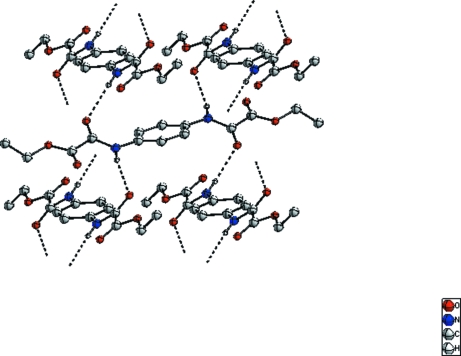
The hydrogen-bonded network in (I). Hydrogen bonds are indicated by dashed lines. All H atoms except the H atoms of imido N atoms have been omitted for clarity.

## supplementary crystallographic information

### Comment

Oxamido ligands were extensively investigated owing to their special biological properties and application prospects (Nonoyama *et al.*, 1982). However, the ligands of oxalamic acid ethyl ester were rarely reported, in which ester group can stabilize the final compounds by producing complexes with main group and transition metals (Rios-Moreno *et al.*, 2003). In this work, the title compound, (I), was characterized by XRD single-crystal diffraction, element analysis and IR.

In the molecule (Fig. 1), the bond lengths containing O and N atoms are all consistent with corresponding values observed in similar systems (Navarro *et al.*, 1998; Hashmi *et al.*, 2004). There exists an intermolecular hydrogen bond involving the carboxamide O atom and the carboxamide N atom with a distance of 3.030 (3) Å, which are in agreement with those found in related compounds. Simultaneously, the molecule units are assembled into two dimensional structure with the intermolecular hydrogen-bond interactions.

### Experimental

The synthesis method of the title compound is according to the previous literature method (Pardo *et al.*, 2003). Colorless single crystals suitable for experiments were obtained from a methanol solution. Elemental analysis calculated for C_14_H_16_N_2_O_6_: C 54.54, H 5.23, N 9.09%; found C 54.52, H 5.22, N 9.08%. IR data: 3249 cm^-1^ (m, N—H), 1682 cm^-1^ (s, C=O).

### Refinement

The H atom attached to the N atom was located in a different Fourier map and refined freely. Other hydrogen atoms were positioned geometrically with bond lengths C—H = 0.93–0.97 Å, and allowed to ride on their parent atoms, with *U*_iso_(H) = 1.2*U*_eq_(aromatic C) or 1.5*U*_eq_(methyl C).

### Crystal data

**Table:**

C_14_H_16_N_2_O_6_	*F*_000_ = 324
*M**_r_* = 308.29	*D*_x_ = 1.396 Mg m^−^^3^
Monoclinic, *P*2_1_/*c*	Mo *K*α radiation λ = 0.71069 Å
Hall symbol: -P 2ybc	Cell parameters from 2859 reflections
*a* = 11.328 (5) Å	θ = 2.2–27.0º
*b* = 7.769 (5) Å	µ = 0.11 mm^−^^1^
*c* = 8.372 (5) Å	*T* = 295 (2) K
β = 95.566 (5)º	Sheet, colorless
*V* = 733.3 (7) Å^3^	0.3 × 0.2 × 0.1 mm
*Z* = 2	

### Data collection

**Table:**

Bruker APEXII CCD area-detector diffractometer	1285 independent reflections
Radiation source: fine-focus sealed tube	1075 reflections with *I* > 2σ(*I*)
Monochromator: graphite	*R*_int_ = 0.017
*T* = 295(2) K	θ_max_ = 25.0º
ω scans	θ_min_ = 1.8º
Absorption correction: multi-scan(*SADABS*; Sheldrick, 2003)	*h* = −12→13
*T*_min_ = 0.975, *T*_max_ = 0.989	*k* = −9→8
5037 measured reflections	*l* = −9→9

### Refinement

**Table:**

Refinement on *F*^2^	Secondary atom site location: difference Fourier map
Least-squares matrix: full	Hydrogen site location: inferred from neighbouring sites
*R*[*F*^2^ > 2σ(*F*^2^)] = 0.048	H atoms treated by a mixture of independent and constrained refinement
*wR*(*F*^2^) = 0.136	*w* = 1/[σ^2^(*F*_o_^2^) + (0.0715*P*)^2^ + 0.3357*P*] where *P* = (*F*_o_^2^ + 2*F*_c_^2^)/3
*S* = 1.00	(Δ/σ)_max_ = 0.022
1285 reflections	Δρ_max_ = 0.33 e Å^−^^3^
104 parameters	Δρ_min_ = −0.19 e Å^−^^3^
Primary atom site location: structure-invariant direct methods	Extinction correction: none

### Special details

**Table:**

Geometry. All e.s.d.'s (except the e.s.d. in the dihedral angle between two l.s. planes) are estimated using the full covariance matrix. The cell e.s.d.'s are taken into account individually in the estimation of e.s.d.'s in distances, angles and torsion angles; correlations between e.s.d.'s in cell parameters are only used when they are defined by crystal symmetry. An approximate (isotropic) treatment of cell e.s.d.'s is used for estimating e.s.d.'s involving l.s. planes.
Refinement. Refinement of *F*^2^ against ALL reflections. The weighted *R*-factor *wR* and goodness of fit *S* are based on *F*^2^, conventional *R*-factors *R* are based on *F*, with *F* set to zero for negative *F*^2^. The threshold expression of *F*^2^ > σ(*F*^2^) is used only for calculating *R*-factors(gt) *etc*. and is not relevant to the choice of reflections for refinement. *R*-factors based on *F*^2^ are statistically about twice as large as those based on *F*, and *R*- factors based on ALL data will be even larger.

### Fractional atomic coordinates and isotropic or equivalent isotropic displacement parameters (Å^2^)

**Table:**

	*x*	*y*	*z*	*U*_iso_*/*U*_eq_	
O1	0.70158 (18)	0.6118 (2)	0.73921 (19)	0.0770 (6)	
O2	0.68604 (16)	0.3705 (2)	0.59603 (19)	0.0759 (6)	
O3	0.82720 (14)	0.44460 (17)	0.97740 (16)	0.0564 (5)	
N1	0.84872 (15)	0.2185 (2)	0.8077 (2)	0.0475 (5)	
H1N	0.826 (2)	0.183 (3)	0.714 (3)	0.058 (6)*	
C1	0.92637 (16)	0.1107 (2)	0.9062 (2)	0.0433 (5)	
C2	0.91424 (18)	−0.0652 (3)	0.8901 (2)	0.0530 (5)	
H2	0.8563	−0.1096	0.8150	0.064*	
C3	1.01346 (18)	0.1763 (3)	1.0168 (2)	0.0515 (5)	
H3	1.0232	0.2947	1.0282	0.062*	
C4	0.80684 (17)	0.3714 (2)	0.8486 (2)	0.0443 (5)	
C5	0.72433 (18)	0.4498 (3)	0.7117 (2)	0.0503 (5)	
C6	0.6271 (4)	0.7022 (4)	0.6127 (4)	0.1143 (14)	
H6A	0.6762	0.7417	0.5314	0.137*	
H6B	0.5689	0.6227	0.5622	0.137*	
C7	0.5702 (4)	0.8389 (6)	0.6711 (5)	0.1472 (19)	
H7A	0.5245	0.8966	0.5846	0.221*	
H7B	0.6275	0.9172	0.7224	0.221*	
H7C	0.5185	0.7995	0.7478	0.221*	

### Atomic displacement parameters (Å^2^)

**Table:**

	*U*^11^	*U*^22^	*U*^33^	*U*^12^	*U*^13^	*U*^23^
O1	0.1129 (14)	0.0571 (10)	0.0539 (9)	0.0175 (9)	−0.0278 (9)	0.0023 (8)
O2	0.0842 (12)	0.0826 (12)	0.0549 (10)	0.0192 (9)	−0.0235 (9)	−0.0186 (9)
O3	0.0795 (10)	0.0479 (8)	0.0392 (8)	0.0048 (7)	−0.0080 (7)	−0.0032 (6)
N1	0.0564 (10)	0.0515 (10)	0.0332 (9)	0.0021 (7)	−0.0034 (7)	−0.0044 (7)
C1	0.0476 (10)	0.0482 (11)	0.0338 (9)	0.0006 (8)	0.0026 (8)	−0.0021 (8)
C2	0.0536 (11)	0.0516 (12)	0.0507 (12)	−0.0030 (9)	−0.0110 (9)	−0.0099 (9)
C3	0.0556 (11)	0.0422 (11)	0.0545 (12)	−0.0030 (9)	−0.0054 (9)	−0.0054 (9)
C4	0.0523 (10)	0.0454 (10)	0.0346 (10)	−0.0041 (8)	0.0012 (8)	0.0024 (8)
C5	0.0558 (11)	0.0541 (12)	0.0400 (11)	0.0022 (9)	0.0000 (9)	−0.0005 (9)
C6	0.176 (4)	0.089 (2)	0.0665 (17)	0.046 (2)	−0.047 (2)	0.0065 (16)
C7	0.162 (4)	0.172 (4)	0.102 (3)	0.092 (3)	−0.017 (3)	0.032 (3)

### Geometric parameters (Å, °)

**Table:**

O1—C5	1.310 (3)	C2—H2	0.9300
O1—C6	1.467 (3)	C3—C2^i^	1.379 (3)
O2—C5	1.193 (2)	C3—H3	0.9300
O3—C4	1.221 (2)	C4—C5	1.533 (3)
N1—C4	1.336 (3)	C6—C7	1.358 (5)
N1—C1	1.419 (2)	C6—H6A	0.9700
N1—H1N	0.85 (2)	C6—H6B	0.9700
C1—C2	1.379 (3)	C7—H7A	0.9600
C1—C3	1.383 (3)	C7—H7B	0.9600
C2—C3^i^	1.379 (3)	C7—H7C	0.9600
			
C4—N1—C1	126.25 (17)	C7—C6—O1	111.9 (3)
C4—N1—H1N	116.1 (15)	C7—C6—H6A	109.2
C1—N1—H1N	117.6 (15)	O1—C6—H6A	109.2
C5—O1—C6	116.2 (2)	C7—C6—H6B	109.2
O3—C4—N1	126.80 (18)	O1—C6—H6B	109.2
O3—C4—C5	121.63 (18)	H6A—C6—H6B	107.9
N1—C4—C5	111.56 (16)	C1—C2—C3^i^	121.15 (19)
C2—C1—C3	119.26 (18)	C1—C2—H2	119.4
C2—C1—N1	118.55 (17)	C3^i^—C2—H2	119.4
C3—C1—N1	122.19 (18)	C6—C7—H7A	109.5
O2—C5—O1	125.2 (2)	C6—C7—H7B	109.5
O2—C5—C4	123.3 (2)	H7A—C7—H7B	109.5
O1—C5—C4	111.49 (17)	C6—C7—H7C	109.5
C2^i^—C3—C1	119.58 (19)	H7A—C7—H7C	109.5
C2^i^—C3—H3	120.2	H7B—C7—H7C	109.5
C1—C3—H3	120.2		

Symmetry codes: (i) −*x*+2, −*y*, −*z*+2.

### Hydrogen-bond geometry (Å, °)

**Table:**

*D*—H···*A*	*D*—H	H···*A*	*D*···*A*	*D*—H···*A*
N1—H1N···O2	0.85 (2)	2.30 (2)	2.701 (3)	109.0 (19)
N1—H1N···O3^ii^	0.85 (2)	2.21 (3)	3.030 (3)	161 (2)

Symmetry codes: (ii) *x*, −*y*+1/2, *z*−1/2.
